# *dms-viz*: Structure-informed visualizations for deep mutational scanning and other mutation-based datasets

**DOI:** 10.1101/2023.10.29.564578

**Published:** 2023-11-01

**Authors:** William W. Hannon, Jesse D. Bloom

**Affiliations:** 1 Molecular and Cellular Biology Graduate Program, University of Washington, Seattle, WA 98109; 2 Basic Sciences and Computational Biology, Fred Hutchinson Cancer Research Center, Seattle, WA 98109; 3 Department of Genome Sciences, University of Washington, Seattle, WA 98109; 4 Howard Hughes Medical Institute, Seattle, WA 98109

## Abstract

Understanding how mutations impact a protein’s functions is valuable for many types of biological questions. High-throughput techniques such as deep-mutational scanning (DMS) have greatly expanded the number of mutation-function datasets. For instance, DMS has been used to determine how mutations to viral proteins affect antibody escape (Dadonaite et al. 2023), receptor affinity (Starr et al. 2020), and essential functions such as viral genome transcription and replication (Li et al. 2023). With the growth of sequence databases, in some cases the effects of mutations can also be inferred from phylogenies of natural sequences (Bloom and Neher 2023) (Figure 1).

The mutation-based data generated by these approaches is often best understood in the context of a protein’s 3D structure; for instance, to assess questions like how mutations that affect antibody escape relate to the physical antibody binding epitope on the protein. However, current approaches for visualizing mutation data in the context of a protein’s structure are often cumbersome and require multiple steps and softwares. To streamline the visualization of mutation-associated data in the context of a protein structure, we developed a web-based tool, dms-viz. With dms-viz, users can straightforwardly visualize mutation-based data such as those from DMS experiments in the context of a 3D protein model in an interactive format. See https://dms-viz.github.io/ to use dms-viz.

## Statement of Need

We wanted dms-viz to provide the following functionalities:

1. **Provide structural context**: The main objective of dms-viz is to simplify the process of visualizing mutation data with structural context by superimposing mutation measurements on a 3D protein structure. Additionally, it provides extensive control over the visual representation of the 3D structure.
2. **Accommodate diverse data types**: Although analyzing DMS data is a key goal of dms-viz, there are many types of mutation data. The tool can handle diverse data types via a command line interface that simplifies the process of converting data into a common format for analysis.
3. **Display multiple conditions**: With dms-viz, multiple experimental conditions can be visualized concurrently, facilitating comparisons. Researchers can, for instance, easily visualize deconvolved antibody binding footprints from polyclonal sera (Yu et al. 2022).
4. **Maximize customizability**: Every dataset has specific needs for visual representation. Recognizing this, dms-viz offers a high level of customizability. Users can tailor filters, which are important for navigating large and possibly noisy datasets, and tooltips, ensuring that the nuances of their data are clear.
5. **Create compact interactive visualizations**: Interactive visualizations promote effective communication. dms-viz creates compact plots that can be incorporated into HTML presentation slides (e.g., https://slides.com/).
6. **Share findings with ease**: Users of dms-viz can generate shareable URL links for a customized visualization view. They can also save and share the JSON specification files created by the command line interface, ensuring that data can be accessed easily.
7. **Preserve data privacy**: dms-viz allows users to analyze proprietary and sensitive data by supporting local upload. This means researchers can view and analyze their confidential structures and datasets without the requirement to store them in a public repository.

Our group previously created a tool called dms-view (Hilton et al. 2020) that has some of the functionalities listed above. However, we designed dms-viz to be more customizable and comprehensive to handle a wider diversity of experimental designs and questions.

## Design and Usage

**Figure 2:**
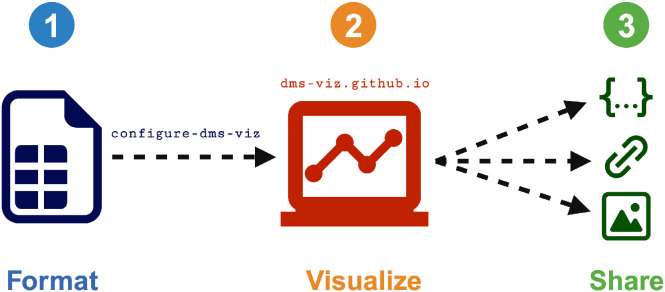
Using dms-viz involves three components. (1) The user formats their data using the command line tool configure-dms-viz. (2) The user takes the resulting JSON specification file and uploads it to dms-viz.github.io. (3) The user can choose to either share the JSON file, host the JSON file and generate a shareable URL link, or export static images.

Using dms-viz involves three components. First, using the command line tool configure-dms-viz, available as a Python package on PyPI (https://pypi.org/project/configure-dms-viz/), the user formats their data into a JSON specification file. Then, the user uploads this specification file to dms-viz.github.io, a web-based interface written in Javascript, D3.js, and NGL.js (Rose et al. 2018). Finally, the specification file can either be shared directly or hosted remotely to generate a shareable URL link (Figure 2).

**Figure 3:**
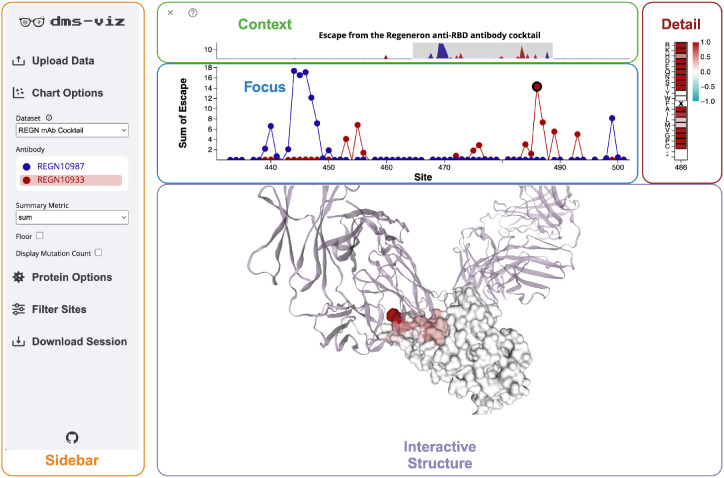
dms-viz provides a compact interface for exploring mutation-associated data. The visual component of dms-viz contains a line/point plot that shows a summary of the mutation-metric at all sites, in this case, mutation-escape from the constituents of a therapeutic antibody cocktail measured by DMS of the SARS-CoV-2 receptor binding domain (RBD) (Starr et al. 2021). The user can zoom into specific regions of interest while maintaining context of the whole dataset using the context plot. Additionally, users can click on points in the focus plot to get details on every mutation for each site in the detail plot. Finally, sites that are selected on the focus plot by dragging are shown on the interactive protein structure colored by the summary statistic. In this example, the structure shown is the SARS-CoV-2 RBD bound to both antibodies in the therapeutic cocktail (PDB: 6XDG). A collapsible sidebar is used to configure the visualization and select the condition on the interactive protein structure. By collapsing out of view, the sidebar makes the visualization an optimal size for integrating into online platforms like websites and HTML presentation slides.

Upon uploading the specification file to dms-viz, users will see a visualization composed of four components, as illustrated in Figure 3.

1. ***Context* plot**: Located at the top of the visualization, this component allows users to zoom into specific sites on the *Focus* plot while maintaining an overview of the entire dataset.
2. ***Focus* plot**: This plot shows a summarized view of the user’s data. Every measured protein site is represented as a point providing a summary statistic of the effects of mutations at that site, and adjacent sites are connected with lines.
3. ***Detail* heatmap**: If the user is interested in the measurements for every mutation at a site, they can click on that site in the *Focus* plot. This will populate a heatmap with each individual mutation measurement at that site.
4. ***Interactive* structure**: When the user wants structural context for a given set of sites, they can drag a brush over the corresponding points in the *Focus* plot. This action will highlight those sites on an interactive 3D protein model.

To ensure the visualization remains compact, all configuration options are tucked away in a collapsible sidebar. See the documentation at https://dms-viz.github.io/dms-viz-docs/ for more information about how to use dms-viz along with detailed tutorials and examples.

## Examples

### 1. Mapping the neutralization profile of antibodies and sera against HIV envelope

Radford et al. (2023) mapped mutations to HIV envelope (Env) that affect neutralization by polyclonal human serum using a pseudotyping-based deep mutational scanning platform (Radford et al. 2023). One aim of their study was to examine how the sites of escape mutations related to HIV Env’s structure. dms-viz excels in generating these visualizations, especially for intra-experimental comparisons. Using dms-viz, it is possible to show multiple antibody footprints on a single summary plot.

See how dms-viz can be used to interactively visualize datasets with multiple conditions here.

### 2. Using mutation-fitness data to augment structure-guided drug design

Bloom and Neher developed a method to estimate the fitness effects of mutations to all SARS-CoV-2 proteins by analyzing millions of human SARS-CoV-2 sequences (Bloom and Neher 2023). These mutation-fitness estimates are useful for purposes such as attempting to design antiviral drugs that target functionally constrained sites where resistance is unlikely to emerge.

By merging Bloom and Neher’s data with structural views of a viral target like the SARS-CoV-2 main protease (Mpro) in complex with a bound ligand such as MAT-POS-e194df51-1 from the COVID Moonshot project (Boby et al. 2023), dms-viz offers an intuitive way to visualize whether a ligand is targeting a mutationally tolerant binding pocket. Computational chemists can incorporate this information into the design process by screening for compounds that target sites where mutations have negative effects on viral fitness.

See how dms-viz can be used to enhance structure-guided drug design here.

### 3. Exploring the evolutionary potential of the influenza A polymerase PB1 subunit

The influenza RNA-dependent RNA polymerase (RdRp) is essential to viral replication, but little is known about the effects of mutations on RdRp function. To address this limitation, Li et al. (2023) measured the effects of thousands of mutations to the PB1 subunit of the RdRP on the replicative fitness of the lab-adapted influenza strain A/WSN/1933(H1N1) (Li et al. 2023).

dms-viz enables facile visualization of these data in the context of PB1’s structure, and can provide stable URL links for easy sharing and access.

See how dms-viz can provide this dataset as an interactive resource here.

## Conclusion

We designed dms-viz as a practical and user-friendly approach to visualizing mutation-associated data in the context of protein structures. Because dms-viz is capable of handling various data types and has options for both sharing and privacy, it should be applicable to visualization of a wide range of datasets.

## Code Availability

- dms-viz is available at https://dms-viz.github.io/
- The documentation and information about developing dms-viz is available at https://dms-viz.github.io/dms-viz-docs/
- The source code for dms-viz.github.io is available at https://github.com/dms-viz/dms-viz.github.io
- The source code for configure-dms-viz is available at https://github.com/dms-viz/configure_dms_viz

## Figures and Tables

**Figure 1:**
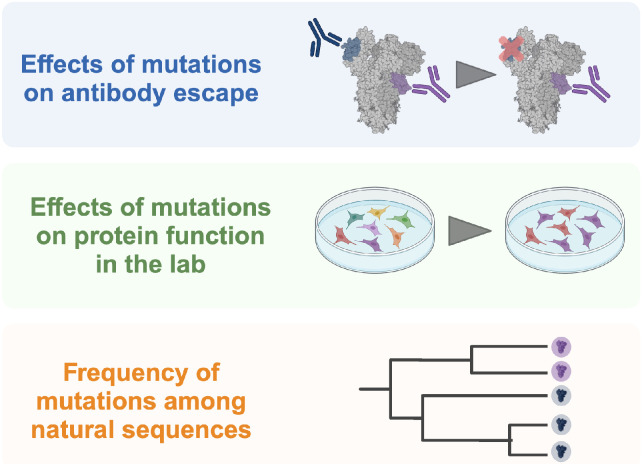
Large mutation-associated datasets are used in a variety of experimental contexts. They can be used to map antibody footprints on viral glycoproteins, assess the impact of mutations on protein function in a laboratory setting, and identify patterns of selection from natural mutation frequencies.
